# Economic Perspectives of Biogas Production via Anaerobic Digestion

**DOI:** 10.3390/bioengineering7030074

**Published:** 2020-07-14

**Authors:** Arpit H. Bhatt, Ling Tao

**Affiliations:** 1 Strategic Energy Analysis Center, National Renewable Energy Laboratory, Golden, CO 80401, USA; Arpit.Bhatt@nrel.gov; 2 National Bioenergy Center, National Renewable Energy Laboratory, Golden, CO 80401, USA

**Keywords:** biogas, anaerobic digestion, waste-to-energy, wet waste, bioenergy, techno-economic analysis

## Abstract

As the demand for utilizing environment-friendly and sustainable energy sources is increasing, the adoption of waste-to-energy technologies has started gaining attention. Producing biogas via anaerobic digestion (AD) is promising and well-established; however, this process in many circumstances is unable to be cost competitive with natural gas. In this research, we provide a technical assessment of current process challenges and compare the cost of biogas production via the AD process from the literature, Aspen Plus process modeling, and CapdetWorks software. We also provide insights on critical factors affecting the AD process and recommendations on optimizing the process. We utilize four types of wet wastes, including wastewater sludge, food waste, swine manure, and fat, oil, and grease, to provide a quantitative assessment of theoretical energy yields of biogas production and its economic potential at different plant scales. Our results show that the cost of biogas production from process and economic models are in line with the literature with a potential to go even lower for small-scale plants with technological advancements. This research illuminates potential cost savings for biogas production using different wastes and guide investors to make informed decisions, while achieving waste management goals.

## 1. Introduction

The demand for energy continues to rise with global population growth and rise in urbanization [1,2]. This has led to increased energy requirements and fossil energy utilization, increasing pollution across the globe [3,4]. With the limited supply of conventional fossil raw materials and their adverse environmental implications on air quality, research efforts on utilizing environmentally friendly renewable alternative sources have gained significant importance [5]. In addition, continuous research coupled with technological developments in this field have helped the investment and deployment of clean energy technologies to surge across the world. One plausible and established waste-to-energy (WTE) option that has been widely adopted is the production of biogas from organic-rich waste streams via anaerobic digestion (AD) process or technology. As waste is an increasing issue worldwide, proper utilization of its energy potential via economically viable and technically feasible technologies can help promote sustainability and meet global renewable energy demand, while limiting risks pertaining to landfilling.

**Figure 1:**
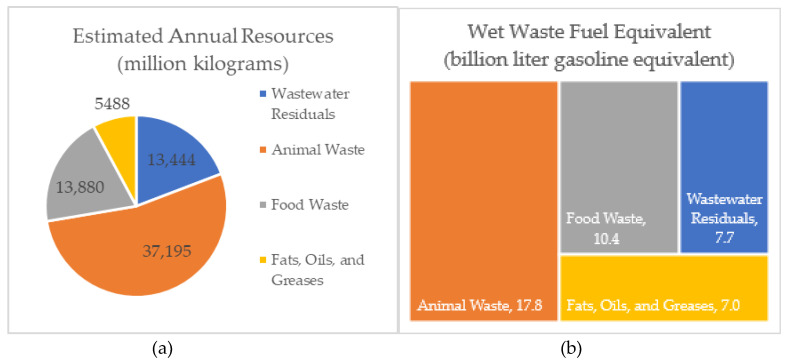
(**a**) Availability of wet wastes in the U.S.; (**b**) Wet waste energy potential in the U.S. [6]. 1 kg = 0.0011 U.S. tons; 1 L = 0.264 U.S. liquid gallons.

The total wet waste feedstocks in the United States (U.S.) present an annual energy resource of 42.9 billion liter gasoline equivalent (11.3 billion gallon gas equivalent [GGE]), which includes wastewater residuals, food waste, animal waste, and fats, oils and greases (FOG) [6]. Conversion of these wet waste feedstocks into useful products, such as biogas (methane [CH_4_] and carbon dioxide [CO_2_]), represents a significant opportunity for additional expansion of transforming under-utilized resources into renewable energy. Different types of organic waste include wastewater treatment (WWT) plant sludge (primary and secondary sludge), agri-food industry waste (part of municipal solid waste including fruit and vegetable by-products, canteen waste, kitchen waste), green waste (waste from shearing of grass, sheets), animal waste (swine, dairy manures), and FOG (animal fats, used cooking oils, restaurant vats for degreasing). Figure 1 shows the wet waste availability in the U.S. along with its energy potential that could be utilized for WTE technologies.

As shown in the U.S. Department of Energy’s (DOE) Bioenergy Technologies Office (BETO) report [6], these wastes are geographically located in areas with high population density. Although the aggregation of wastes provides benefits with lower transportation costs, they are more vulnerable to stringent and cost-intensive disposal regulations owing to landfill constraints. Thus, novel waste management solutions should be considered for this huge waste energy potential that can help with disposal problems. Current and widely applied waste management practices include direct disposal or landfilling and direct combustion or incineration [7,8], which leads to emissions of hazardous pollutants harmful to human health as well as greenhouse gas (GHG) emissions [8,9,10]. Other sustainable practices that are accepted include composting, recycle/reuse of organic matter, and animal feed production, but yield low energy [9]. Moreover, many waste processing technologies are tailored to a specific family of feedstock that has significant compositional, temporal, and geographic variability. Thus, for sustainable energy production, there is a global need to adopt technologies that can utilize a wide variety of environmentally friendly feedstocks with high energy density.

A widely popular and cost-effective technology currently employed at several WWT facilities is AD, which effectively treats a variety of biodegradable streams rich in organic carbon, such as sludge, food waste, yard waste, wood, process residues, animal manure [11], algae biomass from sludge obtained from phototrophic recovery [12,13], etc., and convert them into CH_4_ containing biogas. Biogas contains 40–75% CH_4_ and 15–60% CO_2_ (by volume), with small amounts of hydrogen (H_2_), nitrogen (N_2_), hydrogen sulfide (H_2_S), oxygen (O_2_), and water (H_2_O). Biogas has a wide variety of applications including as a substitute for natural gas and heating oil, an upgrade for utilization as a transport fuel, and use in the production of heat and electricity using combined heat and power (CHP) technology [14]. Moreover, the odors associated with manures along with emissions of several pollutants (e.g., ammonia, CH_4_, CO_2_, nitrous oxide, and methyl-mercaptanes) can be reduced through use of AD technology, enhancing the agricultural sustainability [15]. Although production of biogas using the AD technology provides an environmentally sustainable approach, there are issues that could affect the process and biogas yields depending on the type of wet waste, further explained in the Discussion section.

This study provides a quantitative analysis on theoretical biogas energy yields and offers viewpoints on both technical and economic perspectives of wet wastes to biogas production. This paper provides a framework to establish the cost of biogas production from AD, utilizing a variety of wet wastes at a wide range of plant scales as compared to costs obtained from the literature. We utilize two process and economic models: (1) Aspen Plus coupled with techno-economic analysis (TEA), and (2) CapdetWorks, to estimate and compare the production cost of biogas in U.S. dollars per gigajoule (USD/GJ). The insights on the current technology status, process challenges, energy yields, and economic potentials are summarized in the study in order to not only provide advancements with waste-to-biogas conversion technologies but also to provide recommendations for optimizing the AD process. This analysis helps illuminate the potential cost savings for biogas production and guide investors and AD developers to make informed decisions.

## 2. Current Practices—Anaerobic Digestion for Biogas Production

A series of biological processing steps with the core conversion using the AD technology, converts the complex organic matter in waste products to simple monomers by using a consortia of microorganisms. The AD technology facilitates organic decomposition and reduces inorganic matter in the absence of O_2_ [16]. These organic materials are converted to final products, which are mainly biogas, digestate (a liquid form in most cases), and a combination of solid and liquid effluents derived from digestate treatments [17]. Biogas can be used to produce electricity and heat, while the effluent liquid rich in crop nutrients is used as agricultural fertilizer depending on the amount of nutrient loading, especially nitrogen [17]. The solid fraction from solid/liquid separation of the digestate can be used as dairy bedding or converted to potting soil mixes. Generally, the feed to the digester is pretreated using different physical operations to reduce maintenance issues. For example, the solids from wastewater primarily include primary and secondary sludge which are grinded, shredded, or screened for efficient operations. Additionally, the accumulation of grits inside the tanks reduces the working volume, which affects biogas production and increases maintenance and cleaning [18]. Therefore, degritting is applied to prevent accumulation, which helps to improve process efficiency as well. Similarly, plastics, stones, and metals are removed from food waste during pretreatment depending on the collection methods.

### 2.1. Steps Involved in AD Process

**Figure 2:**
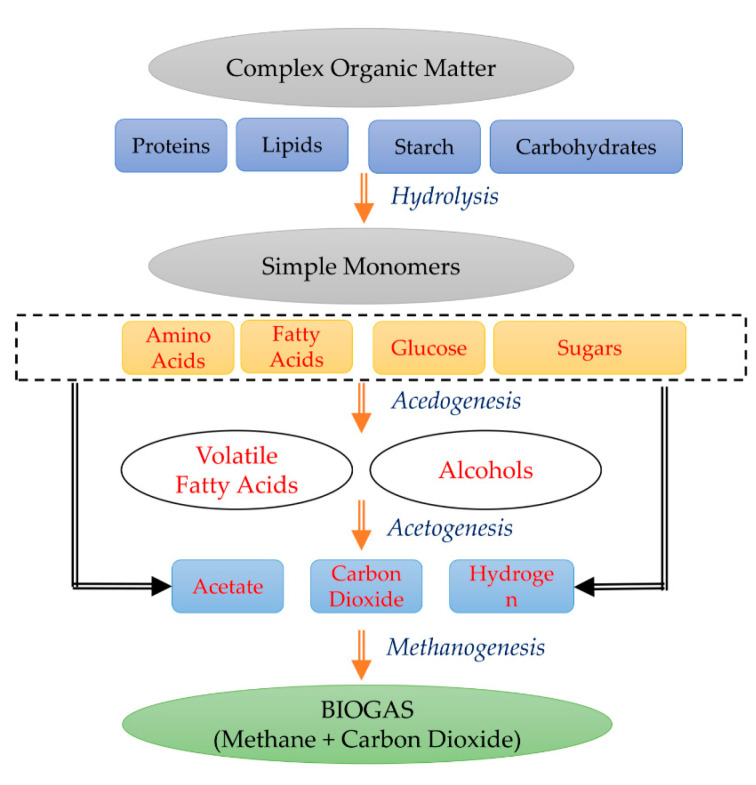
Steps in the Anaerobic Digestion Process.

AD includes four biological steps, as shown in Figure 2 [17]; (1) *Hydrolysis* to breakdown insoluble organic matter to simple monomers via hydrolytic microorganisms. Proteins are converted to amino acids, lipids into fatty acids, starch to glucose, and carbohydrates to monomeric sugars. (2) *Acidogenesis* to convert simple sugars and acids to volatile fatty acids (VFAs) and alcohols via acidogens. This is the fastest step in the AD process which involves complex consortia of bacteria, including bacteriocides, clostridia, bifidobacterial, streptococci, and Enterobacteriaceae [19]. (3) *Acetogenesis* to further convert VFAs to acetate, CO_2_, and H_2_, and (4) *Methanogenesis* to convert acetate and H_2_ to CH_4_ and CO_2_ via methanogenic bacteria. The consortia of microbial populations need to be maintained to stabilize the AD process and increase biogas production efficiency.

### 2.2. Factors Affecting the AD Process

There are several operating factors affecting the production of biogas in the AD process. These mainly include temperature, hydraulic retention time (HRT), organic loading rate (OLR), and pH. Other factors affecting the gas production also include tank volume, feedstock type, feeding pattern, and carbon to nitrogen (C/N) ratio.

Temperature is a critical parameter for the AD process for survival of microbial consortia and to consistently produce biogas, as for each 6 °C decrease (20 °F), the biogas production falls by 50% [17]. Two temperature ranges are most suitable for biogas production—thermophilic and mesophilic. Thermophilic bacteria operate at high temperature conditions (48.9–60.0 °C or 120–140 °F), thus, reducing the retention time to decompose more substrate and produce more biogas. However, these systems are costly to operate as energy is required to maintain higher operating temperature, and they are prone to acidification and are easily influenced by toxins [20,21]. Alternatively, mesophilic bacteria functioning at lower temperatures (32.2–43.3 °C, or 90–110 °F) produce less biogas as compared to thermophilic but are easy to operate, low in investment costs, and more stable towards environmental changes. However, they have poor biodegradability and are susceptible to nutrient imbalance [22,23].

In addition to temperature, HRT also affects biogas production. HRT is the average time (usually, a few days to 40 days, depending on the type of organic waste and digester) feedstocks reside in the digester to decompose based on chemical oxygen demand (COD)/biological oxygen demand. Longer retention times provide enough time for organic matter to degrade depending on the microbial consortia present in the digester at different rates and times. Shorter retention times would inhibit methanogenesis while longer retention times lead to insufficient utilization of components [20,24]. Similarly, the amount of volatile solids (VS) fed to the digester every day (OLR) is also an important parameter affecting biogas yield. Biogas production increases with higher OLR; however, it disrupts the bacterial population, leading to higher hydrolytic bacteria and acidogens. This would lead to lower methanogen population required for biogas production. The literature contains maximum OLRs for various organic feedstocks to avoid irreversible acidification and high biogas yields (9.2 kg VS/m^3^/day for sludge, 10.5 kg VS/m^3^/day for food waste) [25,26], with an optimal range between 1.5 and 6 kg VS/m^3^/day for all waste [15].

pH is another important factor affecting the bacterial activity, and thus, biogas production. Methanogens are highly sensitive to acidic environment (pH < 7), while acidogens are inhibited leading to a rapid increase in methanogens at higher pH levels. The optimal pH for acidogenesis is between pH 5.5 and 6.5 [27], while methanogenesis is most efficient between pH 6.5 and 8.2 [28]. Thus, it is important to maintain pH between 6.5 and 7.5 to sustain an optimal concentration of acidogens and methanogens in the digester for higher biogas yields. Other factors affecting the AD process include type of feedstock for predicting the composition and rate of reaction, tank volume for determining the retention time, and C/N ratio, replicating the amount of nutrient levels in the digester required for AD steps affecting the biogas yield [20].

## 3. Methods

For this analysis, we consider four different types of feed for biogas production—wastewater sludge, food waste, swine manure, and FOG. Table 1 shows the major data assumptions and correlations obtained from the literature for different wastes along with biogas parameters, including mass yield and gas composition analyzed in this study. These assumptions are based on the data obtained from the literature to understand wet organic waste feed characteristics, biogas production, and composition [6,29,30,31,32,33,34,35,36,37,38,39,40,41,42,43,44,45,46,47,48,49,50,51,52,53,54,55].

### 3.1. Feedstock Composition

The feedstock composition can play an important role in the overall yields and process economics of the system. For wastewater sludge, the moisture content varies greatly (>80–99 wt%, mass basis unless otherwise specified) depending on the dewatering techniques employed prior to reaching the AD reactors. Here, we assume that the stream entering the AD reactor has been dewatered to 4% solids content, which is consistent with previous modeled efforts [56]. The ash content of wastewater sludge can also vary greatly depending on the sources, assumed at 7.5% in this study [29]. The nonvolatile solids, other than ash, account for 17.5% of the dry mass such that only 75% of the total dry solids are volatile [30,31]. Proteins have the highest concentration in the wastewater sludge at 24% followed by 18% lipids, 16% fermentable carbohydrates, and 17% of other material set as extractives.

Food waste can vary widely in composition depending on the source and usage. Based on the literature, the moisture content in food waste ranges from 54–87%, ash content ranges from 1–15%, proteins range from 3–44%, lipids range from 4–43%, and carbohydrates range from 10–76% [32,33,34,35,36,37,38,57]. We assume that the food waste entering the AD unit contains 25% solids, with 55% carbohydrates as the largest percent of the biomass on dry basis, followed by 21% lipids, 19% proteins, and 5% ash.

For swine manure, the moisture content is assumed 93% with an ash content of 15% of total solids (TS) [39]. The TS have 37% carbohydrates, 20% proteins, 21% lignin, 4% lipids, and 4% extractives [40]. Swine manure can have a high concentration of nutrients after AD, specifically phosphorous. These biosolids with high nutrient contents can be a revenue generating stream. Further research would be needed to understand the nutrient production amounts and balances to valorize this stream.

Finally, FOGs are predominantly lipids with a small protein and carbohydrate content compared to the other three feedstocks. FOG is a combination of fats, oils, and greases, mainly produced from cooking and the food producing industry. For this study, we consider fat as a primary source of the FOG stream, which is assumed to have 90% moisture, and the dry weight solids have 78% lipids, 7% proteins, and 15% carbohydrates [41].

### 3.2. Biogas Composition and Energy Yield

The composition of CH_4_ and CO_2_ in the biogas produced would vary depending on the waste characteristics and operating conditions of the AD process. A summary of the percentage of initial COD values [44,45,46,47,48,49,50,51,52,53,58,59,60,61,62], COD reduction, and typical biogas production facility scales are shown in Table 1. The fractional conversions of fermentable components are based on the COD reductions from the literature, i.e., 55.5% for sludge, 65% for food waste, 55% for swine manure, and 82% for FOG. It should be noted that depending on the composition and material that comprises the food waste stream, the COD reduction can be as high as 90%. The feedstock composition and the conversion of fermentable components (carbohydrates, proteins, and lipids) into biogas based on waste-specific COD reduction rates forms the basis for estimating the CH_4_ and CO_2_ content in biogas.

The energy yield is another important parameter to estimate the biogas production values for a given amount of waste (on a dry or wet basis). There is a wide range of biogas production values reported in the literature for different types of wet waste. Based on the literature values of biogas production (mass basis), energy content or heating value of the substrate, COD values, and VS and TS in the feed, we estimate the biogas energy yield for sludge, food waste, swine manure, and FOG [6,29,30,41,42,43,44,45,46,47,48,49,50,51,52,53,54,58,59,60,61,62,63,64,65,66,67,68,69,70,71,72]. We also estimate the theoretical energy yield of biogas by dividing the energy content of biogas production (in terms of MJ/kg using the volumetric biogas yield in m^3^/kg and calorific value of biogas in MJ/m^3^) by the feedstock energy inputs (in terms of heat content, MJ/kg) to the process, without accounting for heat loss due to process operation. Refer to Section 4.1 for detailed results on each wet waste feedstock.

### 3.3. Biogas Cost Analysis

#### 3.3.1. Cost Data from Open Literature

To compare the cost of biogas production from various models, we first collect the data from the literature for various AD processes utilizing different types of organic wastes for different plant scales. In addition, we also summarize data for organics present in the feed (COD), VS. and TS content, and biogas production values. We use these values to determine the biogas energy yields for each wet waste feedstock and estimate the biogas production (in GJ) for different feed rates. When data on biogas production rates are not available, we use the theoretical energy yields for each feed type to estimate the costs.

In addition to direct capital costs associated with the AD reactor obtained from literature, we add indirect capital costs, which include additional piping (4.5% of inside battery limits (ISBL]), project contingency (10% of total direct costs [TDC], combination of total installed capital costs and piping costs), and other costs (10% of TDC). We also consider working capital, which is 5% of fixed capital investment (FCI) (combination of TDC and other capital costs). The capital costs are amortized annually considering a 30-year straight line depreciation. We also consider operating expenses, including electricity import, heat, water for the process, and additional nutrients (including ammonia) provided to the AD microbes. Other operating costs include maintenance and property insurance, which is 3% of the plant capital costs and 0.7% of the FCI, respectively. Capital and operating costs are then converted to USD/GJ based on biogas production rates from the literature.

All costs are converted to 2016 USD using the Plant Cost Index from Chemical Engineering Magazine [73], the Industrial Inorganic Chemical Index from SRI Consulting [74], and the labor indices provided by the U.S. Department of Labor Bureau of Labor Statistics [75]. For comparison, we convert the annualized total costs (capital and operating) into USD/GJ based on respective energy yields.

#### 3.3.2. Cost Data from Aspen Plus Simulation

**Figure 3:**
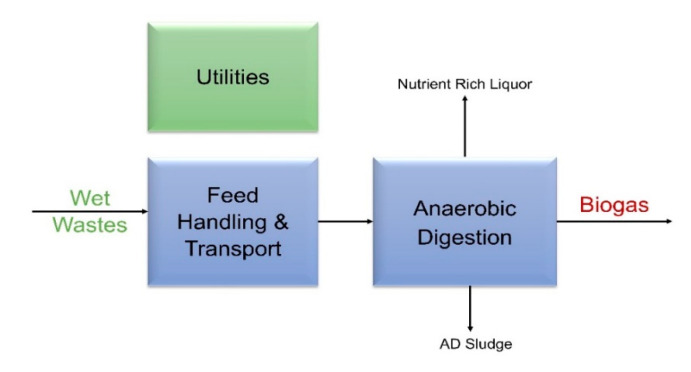
Process Flow Diagram for Modeling AD using Aspen Plus.

We model an AD system using Aspen Plus for four feedstocks at various plant scales (see Table 1) analyzed in this paper. Aspen Plus is a chemical process simulator which includes unit operations to build a process model for simulating complex calculations for integrated batch and continuous processes. First, the process simulation calculates material and energy balances using Aspen Plus [76] based on the block flow diagram shown in Figure 3. Then, an in-house spreadsheet calculates capital and operating expenses for each case. The final effluent out of the AD unit goes through solid-liquid separation to produce AD sludge waste and nutrient rich liquor. Utilities for heat and power requirements are also included in the model. It should be noted that we do not include biogas clean-up in this analysis as most of the literature data do not report this cost.

The design of an AD system can consist of a single stage or of multiple stages with the processing steps of hydrolysis, acidogenesis, acetogenesis, and methanogenesis, separated or combined. There are two types of AD digesters. Mesophilic digesters operate at a lower temperature as compared to thermophilic digesters. The digester performance depends greatly on reactor configuration and total vs. or total percentage COD in the biosolids. The loading rate can be calculated using Equation (1) [77], while sizing of the digesters is based on the solids retention time and the loading rate of the vs. into the system.

(1) $$Loading\;rate\left(\frac{mgCOD}{m^{3}*day}\right)=\frac{organic\;mater\left(\frac{mgCOD}{m^{3}}\right)*Flow\;rate\left(\frac{m^{3}}{day}\right)}{operating\;volume\left(m^{3}\right)}$$

In addition, the stoichiometric reactions modeled in the AD reactor are shown in Equations (2)–(4). The production of biogas from Aspen simulation is based on the fractional conversion of carbohydrates, proteins, lipids, and other components based on defined COD reductions in Table 1.

(2) $$Carbohydrates+H_{2}O\;\to 3\;CH_{4}+3\;CO_{2}$$

(3) $$Proteins+0.591\;H_{2}O\;\to 0.028\;AD\;Sludge+0.4375\;CH_{4}+0.262\;NH_{3}+0.01\;H_{2}S+0.4225\;CO_{2}$$

(4) $$Lipids+23.6\;H_{2}O+1.452\;NH_{3}\to 1.452\;AD\;Sludge+36.37\;CH_{4}+13.37\;CO_{2}$$

We utilize the values of VS. loading rate from the literature to maximize biogas production. We use vs. loading of 0.95 kg VS/m^3^ AD/day for wastewater sludge [56], while a higher value of 4.7 kg VS/m^3^ AD/day is used for food waste because of the high solids content. For swine manure, the vs. loading rate is 1.5 kg VS/m^3^ AD/day [70], while vs. loading for FOG is 3.5 kg VS/m^3^ AD/day [78,79]. Then, the total volume needed for AD is calculated based on both the hydraulic retention time and the total inlet feed flow rate. An in-house excel model is created to import mass and energy data from the process model which are then used to size and cost the AD reactor.

In addition to the AD reactor, we consider the costs of additional capital equipment including biogas blowers, feed screws, coolers, pumps, holding tanks, mixers, and heat exchangers to include other components of the AD system. We follow a similar methodology as described in the previous section to determine the annualized capital and operating costs considering a straight 30-year depreciation to estimate the biogas production costs in USD/GJ.

#### 3.3.3. Cost Data from CapdetWorks

**Figure 4:**
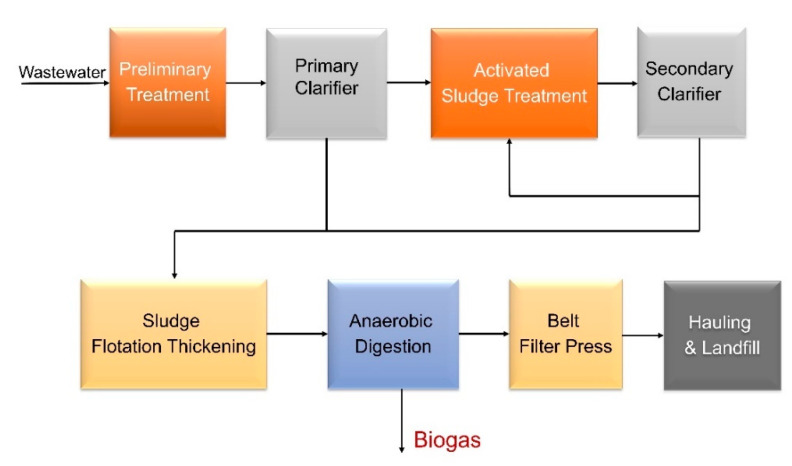
Process Flow Diagram of a WWT Facility as Modeled in CapdetWorks.

**Figure 5:**
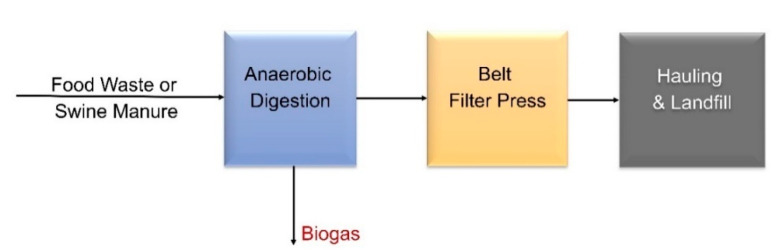
Process Flow Diagram of an AD Facility Converting Food Waste or Manure as Modeled in CapdetWorks.

Like Aspen Plus applied for chemical processes, CapdetWorks is a wastewater industry standard that has been used for various analyses by the U.S. Environmental Protection Agency (EPA) [80,81]. It is an effective software that has a built-in “what if” scenario (unlike Aspen) that performs sensitivity analyses much faster and more flexibly while providing accurate results. CapdetWorks provides two ways to define inlet streams—one is to use the defined wastewater inlet stream from the model, and the other is to use a defined biosolids feed stream (typically applied to food, manure wastes). When using the wastewater sludge, the stream has a low solids and COD content; thus, the process undergoes several dewatering steps to reach the AD unit. Although we model the full wastewater facility with dewatering steps in CapdetWorks (as shown in Figure 4), only the cost of the AD unit is considered as part of this analysis. The food waste and swine manure are fed directly into the AD, as shown in Figure 5.

Though CapdetWorks requires few variable inputs, it is difficult to obtain cost, biogas yields, and sizing values for FOGs because of its limitations to vary COD/VS values. Thus, we do not model FOG in this simulation software. To be consistent with other models, we consider additional piping (4.5% of ISBL), project contingency (10% of TDC), other costs (10% of TDC), and working capital (5% of FCI), in the CapdetWorks economic analysis. The capital costs are depreciated straight-line in a 30-year period, while the amortized operating costs are directly obtained from the model. It is important to note that Aspen Plus simulation software has the ability to perform complex calculations for batch and continuous operations for any type of chemical facility while CapdetWorks is a wastewater treatment industry standard software which can also be used for simulation of biosolids processes.

## 4. Results

### 4.1. Biogas Composition and Energy Yields

Our results suggest that the biogas composition for wastewater sludge and food waste are 60% and 56% methane, respectively, while the methane content of the biogas is 53% for swine manure and 70% for FOG. The deviation of the biogas composition is due to compositional distribution of lipids, carbohydrates, proteins, and other fermentable components in the feed. Although swine manure has low CH_4_ content at 53%, which is most likely due to the higher amount of unfermentable components such as lignin and ash when compared with other wastes, there are several literatures that show CH_4_ content in swine manure to be typically around 65–75%, mainly due to several removal practices followed by the facilities [15,82]. Moreover, co-digesting swine manure with other wastes such as winery wastewater and vegetable processing wastes could increase CH_4_ content to 64% and 69%, respectively, depending on operating conditions [83,84]. Li et al. [85] also showed methane content in excess of 62% when co-digesting swine manure with cow dung in dry AD process. FOG, on the other hand, produces a biogas product with 70% CH_4_, likely due to the high lipids content and zero ash and lignin in the feed. Co-digestion of FOG with other wet wastes (sludge or food waste) would likely reduce the CH_4_ content back to ~60% or lower depending on the co-digestion ratio and COD digestibility.

**Figure 6:**
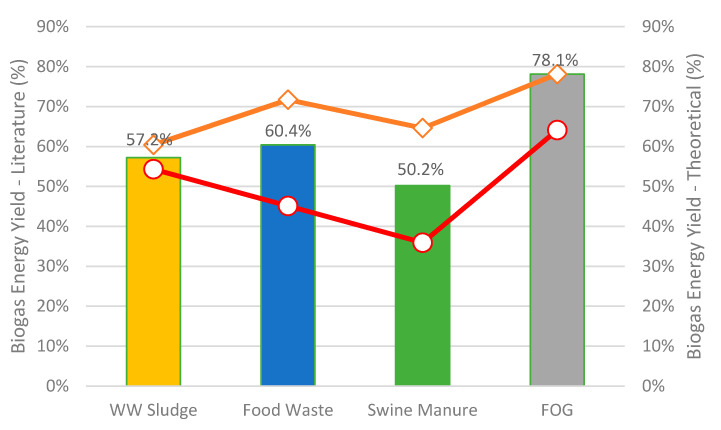
Biogas Energy Yield (%) from Wet Waste Feedstocks. The bar chart shows the theoretical values of energy yield from different wastes while the line chart shows the range of energy yields from the literature.

Figure 6 shows the theoretical biogas energy yields for different wet wastes as compared to range of the literature values.

As mentioned previously, the percentage energy yields shown in the figure are estimated by the energy content of biogas divided by the feedstock energy content to the process. For example, based on the biogas yield of 0.65 m^3^/kg TS (646 m^3^/t TS, as reported in Table 1) of food waste [86], energy content of 24.2 MJ/kg TS (20.8 MMBtu/dry ton) for food waste [87], and calorific value of 22.6 MJ/m^3^ (21,422 Btu/m^3^ at 60% CH_4_) for biogas, the theoretical biogas energy yield is estimated to be 60.4%. This methodology is utilized for each feedstock and the numbers are compared with those obtained from the literature to estimate the range. As shown in Figure 6, the energy yield for wastewater sludge ranges from 54–60%, while it ranges from 45–72% for food waste. Likewise, the energy yield for swine manure ranges from 36–65%, while for FOG, it is 64–78%, with fat as the source component. Thus, the energy yield for FOG is highest at 78.1% and is lowest for swine manure at 36%, among all the wastes. This is because of the lower biogas production from swine manure as compared to other wastes, due to the high quantity of non-fermentable components (e.g., ash) in the feed.

It should be noted that the biogas energy yields from the literature for several wastes are estimated to be higher than theoretical values because of high methane production through pretreatment technologies utilized prior to AD, use of inoculum to increase fermentable composition, or variation of operation parameters (e.g., pH, temperature, loading rate, HRT, etc.,) to boost the productivity. In contrast, the theoretical energy yields are based on biogas composition from fixed COD reduction and without considering any pretreatment technologies and additional operational changes.

### 4.2. Biogas Economics

To provide further insights into the economic potential of producing biogas from wet wastes via the AD process, we estimate the costs of AD units from the literature, Aspen Plus, and CapdetWorks modeling. As mentioned previously, the cost estimates include direct (e.g., equipment costs) and indirect costs (e.g., project contingency, additional piping) along with the working capital, considering a 30-year straight line capital depreciation.

**Figure 7:**
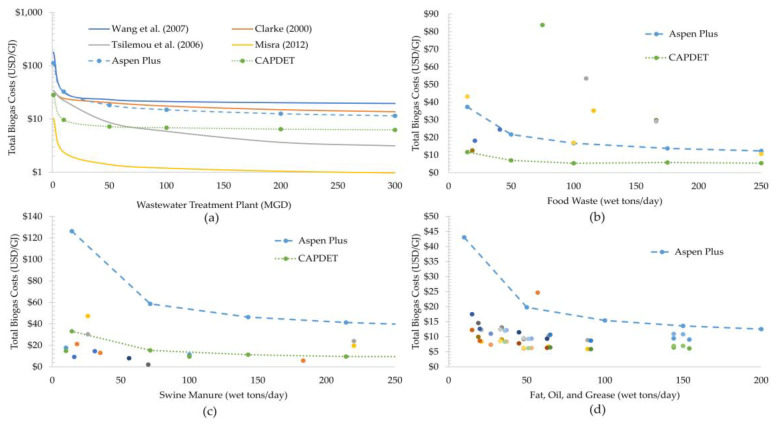
Total Biogas Production Costs in USD/GJ for AD Processing (**a**) Wastewater Treatment Sludge, (**b**) Food Waste, (**c**) Swine Manure, and (**d**) Fat, Oil, and Grease. Please note that that *y*-axis on Figure 7a is plotted on a log-scale.

Figure 7a–d shows the biogas production costs from several literature data sources [88,89,90,91], Aspen process model [76], and CapdetWorks model (except FOG) for facilities using wastewater sludge (1–300 MGD), food waste (1–250 wet tons/day), swine manure (1–250 wet tons/day), and FOG (1–200 wet tons/day), respectively. As shown from the figure, the cost for biogas production from wastewater sludge decreases with increasing scale, following economies of scale. The cost numbers estimated from Aspen and CapdetWorks simulations are in the middle of the plotted literature results [88,89,90,91], showing good agreement of simulated values with the literature. However, the cost numbers are on the optimistic side considering the recent agreement with literature values at plant scales lower than 10 MGD except for Misra [91]. But the cost numbers are quite off for a couple of the literature works at scales greater than 10 MGD. This is mainly due to varying sludge composition across different WWT facilities, some of which may also include pretreatment technologies to make sludge more attractive for the AD process. In contrast, the modeling aspects only consider a fixed composition of wastewater sludge without considering any pretreatment, which causes the disparity in cost numbers. In addition, the cost advantages due to increased output for large-scale facilities helps reduce the cost of biogas to around ~1 USD/GJ, as shown in Misra [91].

For food waste, the literature data sources [77,92,93,94,95] have a wide range of cost values for AD utilizing food waste. This is mainly because the food waste utilized in literature has different sources, which includes food loss before or after meal preparation, waste after consumption, as well as food discarded in the process of manufacturing, distribution, retail, and food services, for which there is a wide variation in amount of VS and its conversion to biogas. In contrast, the costs of AD producing biogas as estimated from Aspen and CapdetWorks simulations follow a cost curve and are lower than literature values. A potential reason might be the high vs. loading rate assumed in the model for high solid content in the food waste. Moreover, the proximate analysis of different types of food (vegetable, meat, etc.) shows different physical properties that may lead to a wide range of AD costs and biogas yields.

Like wastewater sludge, the cost of biogas production from swine manure from the CapdetWorks simulation generally follows the dotted literature values indicating a very good agreement on what is being simulated in lieu of data found in the literature. The only exception is at lower plant scales (<50 wet tons/day), where simulation results presents a sharp increase in costs. In addition, the cost of biogas production from Aspen Plus simulation is higher compared to literature which may be because of the wide range of moisture content found in literature as opposed to fixed composition assumed in the simulations.

For FOG, the cost data from Aspen Plus are slightly higher than the literature at plant scale greater than 50 wet tons/day which has a wide range of values, while it is significantly higher for small facilities, mainly due to costs representing FOG co-digested or blended with other wastes. Additionally, the economies of scale play an important role when scaling the equipment cost as large-scale facilities have more cost-savings and competitive advantages as compared to small facilities with low production volumes. There are exceptions at large plant scales where the composition of co-digested waste could significantly alter the biogas production values and AD costs. It should be noted that the costs could deviate significantly from the actual FOG-to-biogas conversion cost for any plant scale depending on whether the waste primarily consisted of either FOG and when considering this as the sole source of feedstock. It should be noted that the values estimated from the simulation models are based on fixed composition of fermentable components (as described in Table 1) which may vary significantly from the literature values, producing wide variance in the cost results.

## 5. Discussion

The cost estimates obtained from Aspen Plus and CapdetWorks simulations are preliminary results based on predefined assumptions from the literature. Further technological advances in the AD process can enhance CH_4_ content in biogas, which may potentially drive down the costs for AD to be economic at small plant scales. Some of the developments that have been tested include novel pretreatment operations (e.g., physical, chemical, and biological) to enhance biogas productivity and energy intensity [96]. Physical pretreatment includes mechanical and thermal energy disruption, while chemical pretreatment includes the use of chemical substrates to disintegrate waste for easy downstream operations. Biological pretreatment includes the application of microbial consortium or enzymes to enhance hydrolysis of the waste (mainly applied for biomass) for increasing CH_4_ yield in the subsequent AD reactor. Another development is the co-digestion of wet waste with high organic feedstocks, which aims to balance the nutrient content and reduce the negative effects of toxic compounds on the process that ultimately increase biogas yield [97]. In addition, the optimization of operating parameters (e.g., improving COD reduction rates based on loading rate, temperature and pH control, etc.) would help enhance AD performance by keeping the system stable and controlled for high biogas selectivity. The configuration of AD reactors can also help alter operating parameters depending on the type of waste. Propositions have been made for novel digester systems including multi-phased digesters to enhance microbial growth in different chambers individually, use of membrane bioreactors, solid state digestion for waste >15% total solids, psychrophilic AD for cold temperature conditions, and integrated AD systems for producing multiple products [82,96]. A detailed analysis on these technologies around the economic and environmental standpoint should be carried out for better evaluating the tradeoffs between biogas yields and cost of adopting these technologies, which may ultimately help compete with natural gas prices.

### Current Uses and Critical Issues Related to Biogas

**Current uses.** Biogas has a wide variety of applications across different industry sectors as mentioned previously. It can be directly used to produce heat and electricity via a CHP system, or it can be upgraded to remove water vapor, hydrogen sulfide, and CO_2_ for use as a natural gas [98,99]. It can also be used as an engine fuel in internal combustion engines or fuel cells for production of mechanical work and/or electricity generation. Biogas can also be used as a fuel for agricultural pumps depending on the requirements [100], or can be directly upgraded to biofuels competing with biomass-based bioethanol and biodiesel production.

Additionally, biogas can be used to make valuable chemical products either through thermochemical or biological pathways. Thermochemical conversion typically begins by oxidizing the methane to form more reactive and, therefore, chemically malleable species. The biological conversion selectively produces specific compounds by taking advantage of the catalytic effect of methanotrophic or methylotrophic bacteria, by consuming methane as a carbon source. Methylotrophic and Methanotrophic bacteria are one class of bacteria, which is characterized by their ability to utilize a variety of different C-1 substrates including methane, methanol, methylated amines, halomethanes, and methylated compounds containing sulfur [101,102,103,104] as the carbon and energy sources [105,106,107,108,109].

There are three groups in methanotrophs, which are Alphaproteobacterial methanotrophs, Gammaproteobacterial methanotrophs, and Verrucomicrobial methanotrophs [110]. These organisms can be genetically engineered to produce a wide variety of chemicals of interest [111]. The main metabolism of methanotrophs is the methane oxidation via methanol to formaldehyde, which serves as an intermediate in catabolism and anabolism, breaking the rather stable C-H bond in methane under ambient conditions [107]. Formaldehyde or methanol is then oxidized to formate, which can either be further oxidized to CO_2_ by a NAD-dependent formate dehydrogenase with the reducing power for methane metabolism [108,112], or serves as a key branch point to the serine pathway for carbon assimilation and catabolism [113,114], then into the tricarboxylic acid cycle.

Despite the potentially attractive cost of biogas, considerable technical challenges exist, specifically on overcoming the gas-liquid mass transport barrier when converting methane biologically. Currently, biological conversion of biogas is still in the early stage of research development as only the production of single cell protein and poly-hydroxy-butyrate from methane has been commercialized. Further targeted research can reveal other financially viable products, which may be of interest to industries.

**Critical issues.** Although biogas has a wide range of applications, there are several problems associated with the production of biogas. If the biogas from the AD process is not handled effectively, the emission of methane in the atmosphere could be hazardous and penalized heavily for GHG emissions. In addition, the overall positive impact of AD in terms of GHG emissions would be diminished if only a small percent of gas is emitted, as the global warming potential of methane is 23 times of CO_2_ [14]. Also, the process utilizes a consortia of microbial population under a specific set of operating conditions, which if not managed properly, can lead to an unstable system and inefficient biogas production [20]. Moreover, the abundance of natural gas has pushed its prices to all-time lows, which makes it difficult for biogas produced from renewable sources to compete on a price basis.

The EPA has recognized the benefits of promoting net/low-carbon fuels derived from biogas. In the recent rulings, the EPA classified many sources of biogas from cellulosic feedstock for transportation fuels as part of the Renewable Fuel Standard (RFS) [115]. Furthermore, the use of biogas under the RFS can improve AD economics by allowing biogas (containing methane as an energy carrier) producers to generate Renewable Identification Numbers (RINs) [115,116]. The current cellulosic RIN credit is approximately USD5.7–USD8.6/GJ (USD6–USD9/MMBtu) [115]. Without these incentives, for producing fuels from biogas, it is harder to be economically sustainable.

The methane percentage in the biogas could be from 40–70% (with remaining of CO_2_) and the market selling price of biogas varies from USD1.4–USD9.5/GJ (USD1.5–10/MMBtu) [117]. The exact composition of biogas produced varies by the composition of organics in the feed, including fats, proteins and carbohydrates, or carbon to O_2_ ratios. This difference in chemical composition leads to the major classes of substrates in biomass having different expected yields and methane content. Lower methane content indicates lower energy yield from the traditional AD process concept, implying energy yield loss on unavoidable CO_2_ production. Therefore, research efforts have been driven towards the production of higher value products (such as short chain organic acids and alcohols [118]) that could challenge traditional process of biogas production as a main driver of the AD process.

## 6. Conclusions

This paper summarizes the technical and economic perspectives of biogas production from wet wastes using detailed analysis of biogas energy yields to show the potential for waste utilization to satisfy growing energy demand. The energy yield can be used in predicting biogas production once the amount of waste (on either dry or wet basis) is known. Based on the total available waste resources and energy density, the total energy resource for biogas production via the AD process is highest for swine manure, followed by food waste, WWT sludge, and FOG.

This paper also provides a review on the economics of current WTE technology as compared to cost estimates obtained from simulation models, with consistent financial assumptions to ensure reasonable comparison across three sources. Cost estimates of biogas production from process simulations are in line with the data from the literature for all wet wastes except food waste, where some level of deviation is observed. This may be due to different sources of food waste (kitchen waste, winery waste, food waste prior and after usage, etc.) with varying compositions leading to a wide range of biogas yields and costs or may be due to variation of facility scales.

The cost of biogas production using AD has a wide range of values for each feedstock mainly due to economies of scale, and the cost variation would be larger if compared among different feedstock types. Moreover, it would be difficult for biogas to compete with the low prices of natural gas around the world. Thus, technological developments are required to increase methane content in biogas to be cost effective and energy competent with natural gas. Although several waste-to-biogas facilities are already in operation, additional research on process parameters such as maximizing the COD reductions for high biogas production rates or increasing methane content would help inspire future AD developments. This research provides recommendations on process challenges and economic potentials that would help the developers and investors make informed decisions prior to construction.

With methane being a main component in biogas, its emissions from the process can be hazardous and would lead to increased GHG emissions in the atmosphere. In addition, the second largest constituent of biogas is CO_2_, which is unusable, diminishing its energy efficiency. Additional sustainability perspectives should be addressed next in comprehensive ways as we did for energy yields and cost aspects.

## Figures and Tables

**Table 1:** Composition of Wet Wastes and Physical Parameters for Biogas Production via Anaerobic Digestion.

Parameters		Wastewater Sludge	Food Waste	Swine Manure	FOG
**Composition (Dry Weight%)**					
Ash		7.5%	5.0%	15.2%	0%
Lipids		18.0%	21.0%	3.8%	78.0%
Proteins		24.0%	19.0%	20.0%	7.0%
Fermentable Carbohydrates		16.0%	55.0%	36.5%	15.0%
Lignin		0%	0%	21.0%	0%
Extractives (all non-fermentable components)		34.5%	0%	3.5%	0%
**Component Parameters**					
Energy Density	MMBtu/t TS	19.5	22.9	17.1	39.0
	MJ/kg TS	20.6	24.2	18.0	41.1
Moisture Content (%)		96%	75%	93%	6–95%
TS (%)		Primary—2–6%	25%	7%	5–94%
		Secondary—2–10%			
COD (mg/L)	Range	47,200–140,000	39,800–350,000	20,600–35,000	92,000–149,000
	Mean	135,711	154,000	28,430	120,500
COD Reduction		55.5%	65.0%	55.0%	82.0%
Biogas Yield	m^3^/t TS	500–600	646	566	1168–1422
	L/kg TS	500–600	646	565	1169–1422
	MMBtu/t TS	11–13	14	12	20–25
	MJ/kg TS	12–14	15	13	21–27
Typical Scale	wet US tons/day, unless noted	1–300 MGD	1–250	1–250	1–200
	wet metric tons/day, unless noted	1–300 MGD	0.9–227	0.9–227	0.9–181
	kg/day, unless noted	3785–1,135,500 m^3^/day	907–227,000	907–227,000	907–181,000

FOG = fat, oil, and grease, MMBtu = million British thermal units, t = metric ton, MJ = megajoule, TS = total solids, COD = chemical oxygen demand, CO_2_ = carbon dioxide, CH_4_ = methane, m^3^ = cubic meter, MGD = million gallons per day, 1 metric ton = 1000 kg, I US ton = 0.907185 metric tons, 1 MGD = 3785 m^3.^
